# Matrix Metalloproteinase 13 Activity is Required for Normal and Hypoxia-Induced Precocious Hatching in Zebrafish Embryos

**DOI:** 10.3390/jdb8010003

**Published:** 2020-01-31

**Authors:** Christopher D. Small, Megan el-Khoury, Ghislain Deslongchamps, Tillmann J. Benfey, Bryan D. Crawford

**Affiliations:** 1 Biology Department, University of New Brunswick, Fredericton, NB E3B 5A3, Canada; C.Small@unb.ca (C.D.S.); Megan.Elkhoury@unb.ca (M.e.-K.); benfey@unb.ca (T.J.B.); 2 Chemistry Department, University of New Brunswick, Fredericton, NB E3B 5A3, Canada; ghislain@unb.ca

**Keywords:** hatching, matrix metalloproteinase 13 (Mmp13), zebrafish, hypoxia, in vivo zymography, stress, developmental trade-off

## Abstract

Hypoxia induces precocious hatching in zebrafish, but we do not have a clear understanding of the molecular mechanisms regulating the activation of the hatching enzyme or how these mechanisms trigger precocious hatching under unfavorable environmental conditions. Using immunohistochemistry, pharmacological inhibition of matrix metalloproteinase 13 (Mmp13), and in vivo zymography, we show that Mmp13a is present in the hatching gland just as embryos become hatching competent and that Mmp13a activity is required for both normal hatching and hypoxia-induced precocious hatching. We conclude that Mmp13a likely functions in activating the hatching enzyme zymogen and that Mmp13a activity is necessary but not sufficient for hatching in zebrafish. This study highlights the broad nature of MMP function in development and provides a non-mammalian example of extra-embryonic processes mediated by MMP activity.

## 1. Introduction

Hatching is a major transition in the life history of all metazoan embryos. Prior to hatch, embryos depend solely on maternal resources loaded into oocytes during oogenesis. The protective membrane surrounding the embryo (i.e., zona pellucida, chorion, or vitelline envelope) must be breached before the maternal yolk endowment is fully consumed or the animal will starve. In mammals, hatching must occur before embryos can implant into the uterine endometrium where they can acquire maternal resources via the placenta [1]. In teleost fishes (and most other metazoans), hatching is the stage at which embryos transition from a sessile and sheltered life to motile and exposed free-living larvae that can move away if conditions are poor. There is a trade-off here between the protection provided by the chorion and the need to begin obtaining nutrients from the environment before maternal supplies are depleted. Embryos are best served by remaining within the sheltered environment of the chorion for as long as possible, while still ensuring they have time and resources available after hatch to avoid starvation.

A key prediction of this hatch-timing compromise hypothesis is that environmental changes affecting metabolic rate and energy utilization should cause predictable shifts in hatching rates. Metabolizing yolk faster (e.g., at high temperature) or less efficiently (e.g., due to hypoxia) should trigger an early hatch. Consistent with this hypothesis, embryos reared at high temperature [2], fluctuating temperature [3], or under mild hypoxia [4] hatch earlier than their respective controls. Interestingly, despite hatching early, larvae raised at higher temperatures are larger at hatch [2], whereas larvae raised under mild hypoxia are smaller at hatch [4], suggesting that hatch timing is regulated by energy utilization and availability rather than metabolic rate alone. The plasticity of the mechanism regulating hatching time in accordance with rearing conditions seems adaptive for environments that may be mildly sub-optimal, but the lack of motility becomes problematic as conditions deteriorate below the threshold at which embryos can compensate metabolically. The biochemical degradation of the chorion by proteases secreted by the hatching gland must be coordinated with the metabolic status of the embryo, and therefore, the mechanism that triggers hatching needs to sense when energy will become limiting under the local environmental conditions.

The link between hypoxia, development, and hatching is particularly relevant to fishes, as many teleost embryos develop in conditions that can be quite hypoxic. For example, salmonids (family Salmonidae) lay their eggs in redds dug into the streambed with dissolved oxygen levels (dO_2_) ranging from ~25% to ~80% air saturation [5,6], and sticky lumpfish (*Cyclopterus lumpus*) eggs are molded into large egg masses with irrigation channels that must be constantly flushed with oxygenated water by guardian males [7,8]. Furthermore, embryonic metabolism generates a local hypoxic zone that extends beyond the chorion and surrounds the embryo. The size and degree of hypoxia in this local zone is affected by water flow, environmental dO_2_, and metabolic rate [9,10]. Exposure to dO_2_ below what DiMichelle and Powers described as a “trigger level” induces precocious hatching in many teleost species [11,12,13]. However, embryos need to develop to a stage at which they are biochemically and physically capable of hatching before this can happen. Robertson et al. [14] identified a developmental threshold at 36 hours post fertilization (hpf) in zebrafish (*Danio rerio*) after which an acute hypoxic exposure (4 h at 0.5% dO_2_ at 28 °C) triggers rapid hatching of the entire clutch of embryos. Because embryos exposed prior to this stage (e.g., at 24 hpf) do not hatch early, Robertson et al. concluded that hatching competence is not attained until roughly 36 hpf. Zebrafish development has been studied extensively in laboratory settings where abiotic conditions can be controlled and dO_2_ levels are typically maintained at or near 100% air saturation, but little is known about the abiotic conditions that zebrafish embryos typically experience in nature. Adult zebrafish tend to inhabit highly vegetated, slow-moving waters prone to eutrophication, such as oxbow lakes and rice paddies [15,16], and early embryos are extremely tolerant of hypoxia and even anoxia [17]. Therefore, it is likely that zebrafish embryos are frequently exposed to hypoxic conditions in their natural environment and would have adaptive characteristics such as the ability to modify hatch timing to escape intolerably hypoxic conditions.

The chorion is a proteinaceous membrane primarily comprised of zona pellucida (ZP) proteins. ZP protein gene copy number varies greatly among fishes driving the evolution of a diverse collection of strategies for producing a fibrous matrix to surround and protect an externally developing embryo. The defining characteristic of these proteins is the ZP domain required for polymerization to form fibrils and matrices [18]. Biochemical degradation of the chorion using pronase reduces the punching force required to pierce the chorion [19,20]. Reduced force is also required to pierce the chorion of post-pharyngula-stage embryos compared to blastula-stage embryos, highlighting the fact that the chorion is modified biochemically (“chorion softening”) before hatching occurs [20]. Enzymatic degradation of this network of ZP proteins by secretion of hatching enzyme (HE), or chorionase, into the chorionic fluid by the hatching gland is a tightly regulated and well-conserved process such that HE can degrade the chorionic proteins in distantly-related species [21,22]. The zebrafish genome encodes 2 HEs (zHE1 and zHE2). zHE1 mRNA is detected as early as 19 hpf and is no longer detected post-hatch, but zHE2 is not expressed at these time points [22,23]. There is evidence of sub-functionalization of HE paralogues in some teleost lineages, but zHE2 is not thought to be relevant for hatching in zebrafish [23]; we will not consider it further. Purified recombinant activated zHE1 degrades ZP proteins from chorions of unfertilized eggs and fertilized embryos at specific cleavage sites in vitro [22], but it remains unclear how the activity of this enzyme is regulated in vivo.

HE is an astacin-like metalloprotease with a zinc-dependent catalytic domain produced by cells of the hatching gland [22,23,24]. As with other metalloproteases, HE has an auto-inhibitory N-terminal propeptide that must be proteolytically cleaved to activate the zymogen [25]. The hatching gland is a cluster of cells that typically reside on the surface of pre-hatch teleost or amphibian embryos. Hatching gland ontogeny has been described in only a few species of teleost and amphibians. Despite having very similar gene expression profiles, the site of differentiation and migration of hatching gland cells varies among taxa [26]. In the hatching gland, HE accumulates gradually in secretory granules as the embryo nears hatch. Both the inactive (zymogen) and active forms of HE are present in isolated secretory granules when extracted in the presence of EDTA, but in the absence of EDTA, only activated HE is detected [27]. Agents that chelate divalent cations such as EDTA inhibit the activity of all metalloproteases, including HE [11,12], by removing the catalytic zinc ion necessary for proteolysis. The observation that inactive pro-HE can be detected in secretory granules isolated in the presence of EDTA led Yasumasu and colleagues [27] to surmise that pro-HE is activated by an EDTA-sensitive protease in the hatching gland, and suggested that HE was likely activated by autocatalysis as they did not detect any other EDTA-sensitive proteases in hatching fluid or hatching gland extracts. Here we identify matrix metalloproteinase 13a (Mmp13a) as an alternative candidate for the “EDTA-sensitive protease” first posited by Yasumasu et al. [27] and argue that the necessity of Mmp13 activity for hatching implies that HE is not activated by autocatalysis.

The matrix metalloproteinases (MMPs) are a highly conserved family of secreted, zinc-dependent endopeptidases that are best known for their ability to degrade and remodel the extracellular matrix (ECM) [28,29]. More recently, it has become clear that ECM remodeling is only one facet of a complex suite of the biological functions in which these proteases play a central role [30,31,32,33]. There are 25 genes encoding MMPs in the zebrafish genome [34]. This number may inflate the apparent degree of diversity of MMPs in fish, as many are duplicates that arose from the ancestral teleost genome duplication [35,36]. However, it is becoming clear that neo- or sub-functionalization has occurred as these paralogues diverged [33]. MMP1, -8 and -13 are the “collagenases” found in mammalian genomes, and all three of these MMPs can efficiently degrade fibrillar collagens [37]. Only orthologues of MMP13 are present in the genome of zebrafish, medaka, and fugu [34], and only Mmp13a is expressed during pre-hatching development in zebrafish. The paralogous Mmp13a and Mmp13b are the only MMPs in the zebrafish genome classified as collagenases [34] which are MMPs known to degrade Type I Collagen [37]. *mmp13b* expression is not detectable during embryogenesis, and we will therefore not consider it further here. *mmp13a* transcription is upregulated by cortisol [38], and Mmp13a activity is increased in situations of oxidative stress [39], making it a likely candidate for protease to function in the hatching mechanism.

Here we show that the ontogeny of Mmp13a during development is spatiotemporally consistent with a role in hatching: it is first detected in the hatching gland just before embryos become hatching competent (24 hpf), accumulates gradually until hatch (48–72 hpf), and is barely detectable in the hatching gland post-hatch (96 hpf). Specific pharmacological inhibition of Mmp13a activity completely blocks hatching under standard rearing conditions and inhibits precocious hatching under hypoxia. Surveying the proteins present in the chorionic fluid reveals widespread proteolysis induced by acute hypoxia at both embryonic stages although this effect is far more pronounced at 36 hpf. Using in vivo zymography, we confirm reports that the chorionic fluid of zebrafish embryos is strongly collagenolytic [40] and demonstrate for the first time that A) this collagenolytic activity is dependent on Mmp13a specifically and B) that this pathway is necessary for hatching in zebrafish. We conclude that hatching is triggered by Mmp13a activity upstream of HE activation and that this trigger is responsive to both developmental timing and environmental stressors, providing a mechanism that implements the hatch-timing compromise.

## 2. Materials and Methods

### 2.1. Animal Husbandry

Zebrafish (Wildtype Tübingen strain) were maintained in flow-through dechlorinated municipal water in the University of New Brunswick Zebrafish Facility in standard 25 × 11 × 15 cm tanks (Pentair Aquatic Ecosystems) at 28 °C on a 14 h:10 h light:dark photoperiod. Adults were fed a standard zebrafish diet (Skretting) twice per day supplemented with *Artemia* once per day. Three males and two females were given 1 h to spawn in 1L breeding tanks (Pentair Aquatic Ecosystems) and embryos were collected 1 h after lights turned on in the morning. Embryos were maintained in Embryo Rearing Medium (ERM: 13 mM NaCl, 0.5 mM KCl, 0.02 mM Na_2_HPO_4_, 0.04 mM KH_2_PO_4_, 1.3 mM CaCl_2_, 1.0 mM MgSO_4_, and 4.2 mM NaHCO_3_, pH 7.4) [19] at 28 °C and staged according to Kimmel et al. [41]. All procedures involving adult animals were approved by the UNB Animal Care Committee, according to the guidelines of the Canadian Council of Animal Care.

### 2.2. Environmental Hypoxia Experiment

Embryos were transferred at 24 or 36 hpf into glass metabolic chambers in which oxygen concentration could be measured using a fiber optic sensor (PreSens Precision Sensing). The chambers were filled with either normoxic ERM (dO_2_ at 100% air saturation, 7.4 mg/L at 28 °C) or hypoxic ERM (dO_2_ at 0.5% air saturation, 0.4 mg/L at 28 °C) generated by bubbling nitrogen gas through ERM while measuring dO_2_ until the desired oxygen concentration was achieved. Embryos were sealed in these chambers for 4 h, with 20 embryos per chamber and 3 replicates per treatment. The %dO_2_ was measured at 30 min intervals to confirm that the volume of the chambers (75 mL) was large enough that embryonic oxygen consumption was negligible. Control chambers with ERM but no embryos were included to monitor background change in %dO_2_, which was also negligible. For perichorionic fluid extractions, the duration of exposure to hypoxia was reduced to 3 h in order to reduce the likelihood of hatching during the treatment. After unsealing the chambers, embryos were transferred back to normoxic ERM at 28 °C for the remainder of the experiment. Hatched embryos were counted and removed every 3 h until 72 hpf.

### 2.3. MMP-13 Protease Inhibitor (Mmp13PI) Experiment

MMP-13 protease inhibitor (Mmp13PI) (4-*N*,6-*N*-bis[(4-fluoro-3-methylphenyl) methyl] pyrimidine-4,6-dicarboxamide) (Santa Cruz Biochem) was dissolved at 10 mg/mL in Dimethyl sulfoxide (DMSO) and stored at −20 °C; experimental concentrations were achieved by diluting this stock in ERM. Vehicle controls were included to control for any potential confounds from the solvent. Experimental concentrations included 20 nM (approximately double the IC_50_ value for human MMP-13) and 100 nM (approximately 10 times the IC_50_). Embryos (20 per treatment with 3 treatment replicates) were reared at these concentrations from 1 hpf until 96 hpf with a change of rearing solution every 24 h. Hatched embryos were counted and removed every 3 h until 72 hpf.

### 2.4. Immunohistochemistry

Embryos were collected at 24, 36, 48, and 72 hpf and fixed in Dent’s fixative (80% methanol, 20% DMSO) overnight at 4 °C, washed thrice in PBS with 0.1% Triton-X100 (PBSTx), and blocked for 8 h in 5% BSA in PBSTx at 4 °C. Primary (anti-Zebrafish Mmp13 (AS-54406, AnaSpec), diluted 1:1000 in blocking buffer) and secondary (Goat anti-Rabbit Alexa488 (ThermoFisher), diluted 1:5000 in blocking buffer) antibody incubations were done overnight at 4 °C on consecutive days. Embryos were washed for 15 min three times with 0.1% PBSTx after each incubation step. Embryos treated identically without the primary antibody incubation exhibited very weak, ubiquitous autofluorescence (data not shown). Confocal stacks were collected using a Leica SP2 confocal microscope, fitted with a 20× 0.7 NA lens. Images were assembled and Z-projected using Fiji [42], and plates were assembled and annotated in Affinity Photo (version 1.7.3, Serif Software, West Bridgford UK).

### 2.5. Molecular Modelling

All molecular modelling was carried out with the Molecular Operating Environment (MOE) drug discovery software version 2016.08.02 (Chemical Computing Group Inc., Montreal, Canada). All molecular mechanics calculations were performed with the Amber10:EHT force field and reaction-field (R-field) solvation. All homology modelling was carried out using MOE and default modelling settings.

#### 2.5.1. Self-Docking Test

The crystal structure of the human MMP-13-inhibitor complexed with the non-zinc binding inhibitor *N*,*N*′-bis(4-fluoro-3-methylbenzyl)pyrimidine-4,6-dicarboxamide was obtained from the Protein DataBank (file id:1XUD.pdb). To test the docking protocol, the inhibitor was deleted from the protein and docked back into the receptor using MOE’s flexible ligand docking facility (MOE_Dock, Placement:Triangle Matcher, Scoring:London dG, 500 poses; Refinement:Induced Fit/GBVI/WSA dG, 10 poses). The best docking pose of the inhibitor (S = −9.48 kcal/mol) was found to be indistinguishable from that in the crystal structure. These docking parameters were retained for all subsequent docking experiments.

#### 2.5.2. Homology Modelling of Zebrafish Mmp13a

Using MOE’s homology modelling module, the primary sequence of zebrafish Mmp13a was read as .pir file. A human MMP-13 protein monomer from the 1XUD.pdb file was used as template. Sequence alignment followed by homology modelling using default MOE settings produced a final homology model that showed excellent backbone superposition to that of human MMP-13 (Root Mean Square Distance: 2.3 Å over 166 residues).

#### 2.5.3. Docking of Inhibitor to Zebrafish Mmp13a Homology Model

Relevant Zn^2+^ and Ca^2+^ atoms from the 1XUD.pdb file were added to the zebrafish homology model. Docking the inhibitor to the Mmp13a homology model produced poses that were significantly displaced from the inhibitor pose of 1XUD. Closer inspection revealed that the Tyr_106_ side-chain of the zebrafish Mmp13a model impeded inhibitor docking in the same pose as that found in 1XUD. Geometry-optimization of residue Tyr_106_ by energy minimization, while fixing all other receptor atoms, rotated the Tyr_106_ side-chain to a much more favorable position. Docking the inhibitor to this corrected zebrafish Mmp13a homology model produced a best pose (S = −9.59 kcal/mol) that was nearly identical to that found in 1XUD.

### 2.6. SDS-PAGE

Chorionic fluid samples were collected manually from pools of 20 embryos per treatment. Immediately after control/hypoxia exposure, and again 3 h after the end of the treatment, 20 embryos from each metabolic chamber were collected onto a chilled depression-slide on ice. Any ERM was removed with a gel-loading pipette, and 10 µL of 10 mM EDTA was added to block protease activity. Embryos were manually dechorionated using #5 watchmaker forceps and the chorions and embryos removed, leaving chorionic fluid and EDTA. This fluid was collected with a gel-loading tip under a dissecting microscope to ensure that any small pieces of chorion were excluded. Samples were transferred to an equal volume of gel loading buffer, quickly mixed, and centrifuged (12000 RCF for 60 s) to remove insoluble debris, and resolved by SDS-PAGE on 10% acrylamide gels at 100 V. Gels were silver stained using a modified version of the protocol described by Sammons et al. (1981) [43].

### 2.7. In Vivo Zymography

Collagenolytic activity in the perichorionic fluid was assayed using in vivo zymography [44,45]. Type I DQ Collagen (Sigma) (1 mg/mL) with (n = 10) or without (n = 10) Mmp13PI (20 nM) microinjected into the perichorionic fluid, and fluorescence images were captured using a Leica MZ205A fitted with a DFC360 FX greyscale low-light camera immediately after injection and again at 1 h after injection. Images were processed identically, using linear histogram adjustment in Fiji [42] such that the dynamic range of the embryos exhibiting the strongest fluorescent signal nearly filled the dynamic range.

### 2.8. Statistical Analysis

All statistical analysis was done in R [46]. Survival curves were generated using Kaplan−Meier survival analysis using hatch as the endpoint rather than survival (hereafter referred to as hatching curves) and compared using the log-rank test with the following packages: ggplot2 [47], survival [48], and survminer [49]. Statistical significance was ascribed at p values less than 0.05.

## 3. Results

### 3.1. Hypoxia Induces Precocious Hatching at 36 hpf

**Figure 1:**
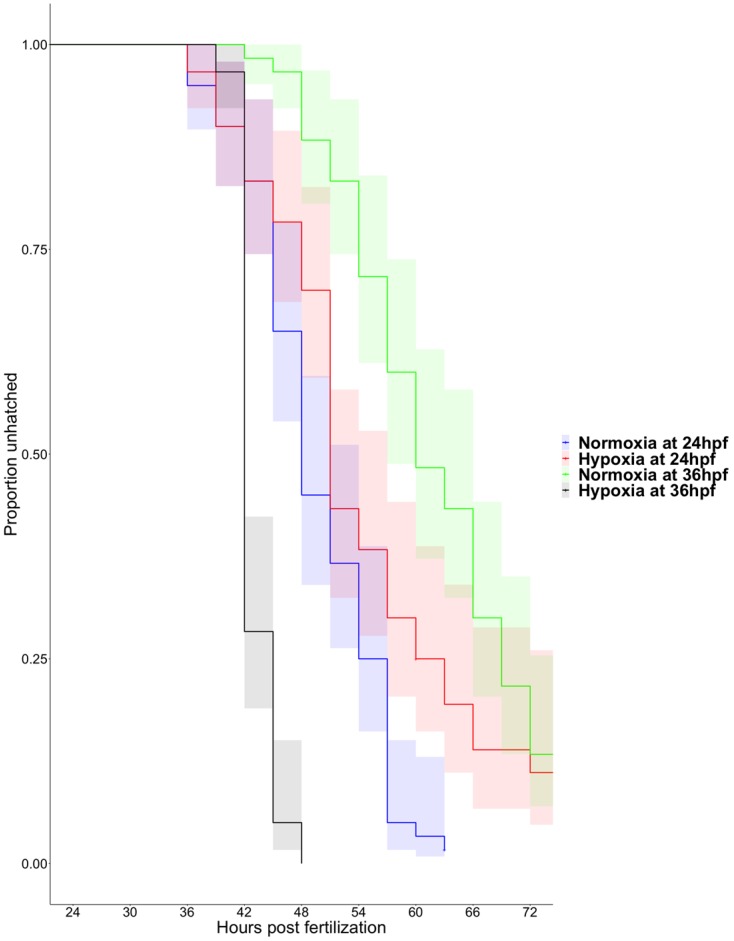
Interactive effect of developmental stage and hypoxia treatment on precocious hatching in zebrafish embryos. Hatching curves with 95% confidence intervals for embryos exposed to hypoxia at 24 and 36 h post fertilization (hpf) and normoxic controls. Hatching analysis indicates significant effects of hypoxia on hatching rate (*n* = 3, 20 embryos/replicate, *p* < 0.0001). Post-hoc pairwise comparisons identify significant differences among all 4 treatment groups; however, the direction of the effect relative to the control is reversed at 36 hpf (*p* < 0.001) compared to 24 hpf (*p* < 0.01).

Time to hatching is quite variable in zebrafish beginning as early as 40 hpf or as late as 75 hpf under our laboratory conditions (Figure 1). Four-hour incubation in severely hypoxic water (0.5% dO_2_, 0.4 mg/L) delays hatching at 24 hpf (*p* < 0.01) but induces a rapid hatching response in 36 hpf zebrafish embryos (*p* < 0.0001) (Figure 1). Reassuringly, the hatching curves of 36 hpf embryos are significantly different from the hatching curves of 24 hpf embryos (*p* < 0.01), as we expected older embryos to be more likely to hatch than younger embryos. Hatching analysis (using standard survival analysis statistics) and pairwise comparison among all groups using the log-rank test indicate that all treatment groups are statistically different from each other, but there is a dramatic reversal in the direction of this effect between 24 and 36 hpf.

### 3.2. Matrix Metalloproteinase 13a Activity is Necessary for Hatching

**Figure 2:**
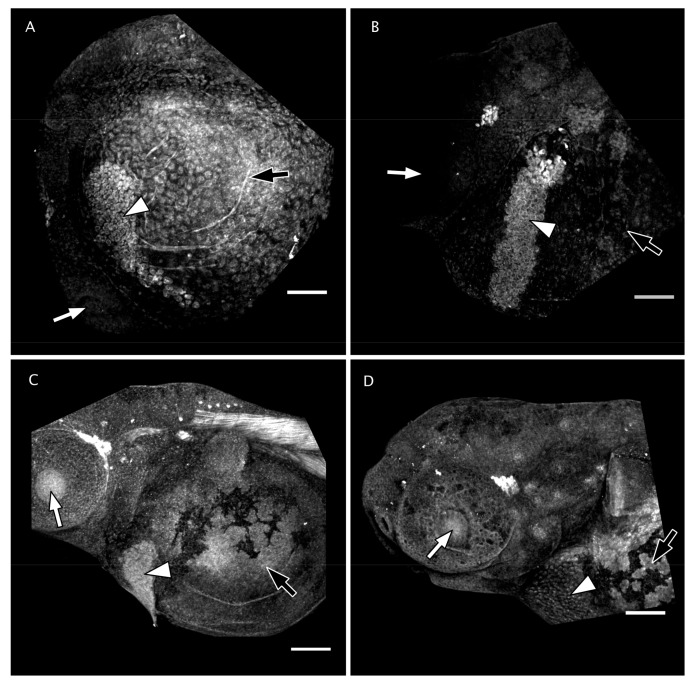
Matrix metalloproteinase 13a (Mmp13a) is abundant in the hatching gland prior to hatching. Projections of confocal stacks through the head and anterior yolk sack of (**A**) 24 hpf, (**B**) 36 hpf, (**C**) 48 hfp, and (**D**) 72 hpf embryos immunofluorescently labeled with anti-Mmp13a. Mmp13a immunoreactivity is strong in the hatching glands (white arrowheads) of 24, 36, and 48 hpf embryos but is greatly diminished in post-hatch 72 hpf embryos. All embryos are oriented anterior to the left, dorsal up, with white arrows indicting the eye and black arrows indicating the yolk sack for reference. Scale bars are 100 µm.

Mmp13a is abundant in the hatching gland of zebrafish embryos before hatching (Figure 2A–C) and is depleted from the hatching gland cells in post-hatch embryos at 72 hpf (Figure 2D). Spatiotemporal correlation of the presence of an enzyme with a biological function does not necessarily indicate a functional role for that enzyme; therefore, we validated a specific pharmacological inhibitor of human MMP-13 for zebrafish Mmp13a to determine if we could use this reagent to block Mmp13a enzymatic activity.

**Figure 3:**
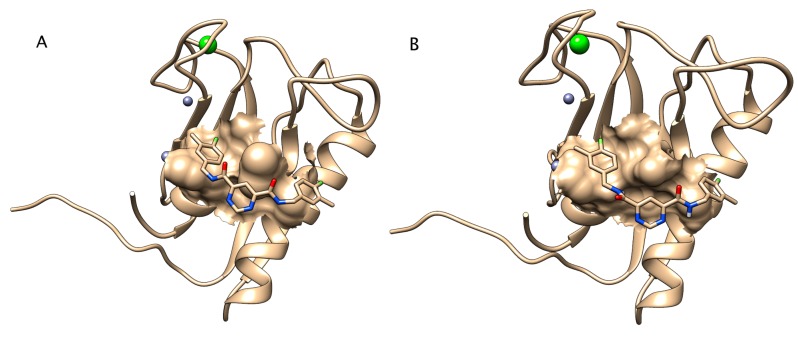
Zebrafish matrix metalloproteinase 13a binds Mmp13 protease inhibitor (PI) with comparable affinity to human matrix metalloproteinase-13. The structural homology model of zebrafish Mmp13a shows excellent backbone superposition with human MMP-13. Mmp13PI docks similarly in the catalytic site of both (**A**) human MMP-13 and (**B**) zebrafish Mmp13a. Zinc and calcium ions are shown as green and greyish blue spheres, respectively.

Homology modeling reveals that the primary sequence of zebrafish Mmp13a can be threaded onto the crystal structure of human MMP-13 with excellent backbone superposition (RMSD: 2.3 Å over 166 residues) (Figure 3). Inhibitor docking simulations using the highly specific human MMP-13 inhibitor [50] successfully docked the inhibitor into the catalytic site of both human and zebrafish Mmp13a with comparable IC_50_ values (8 nM for human compared to 20 nM for zebrafish). Note that this is 500–1000-fold lower than IC_50_ values of this molecule for other metalloproteinases [50].

**Figure 4:**
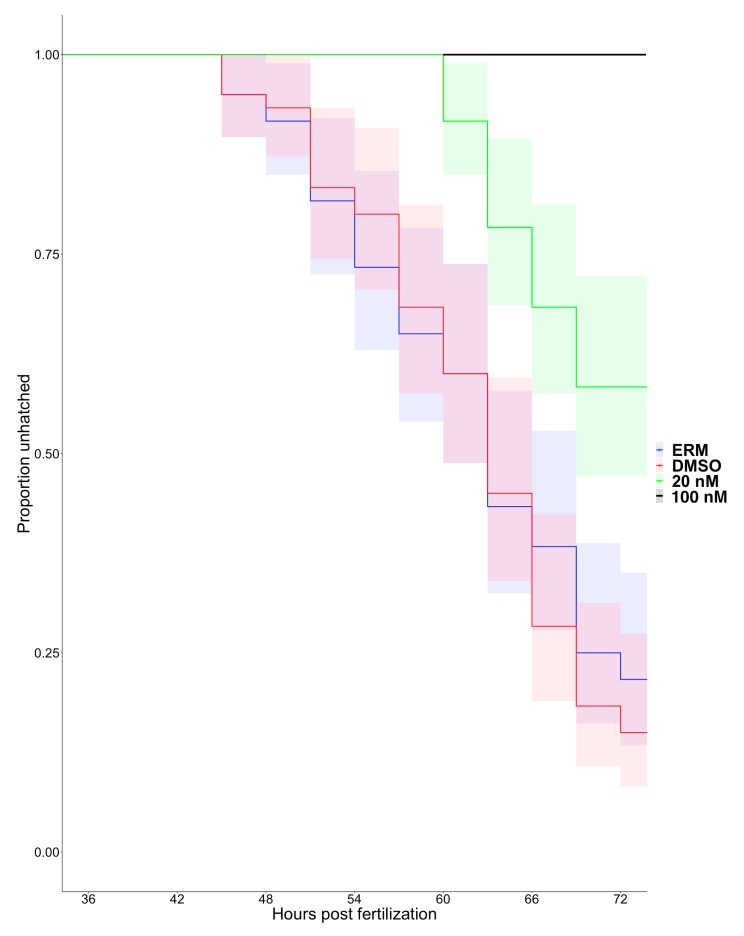
Mmp13a activity is necessary for hatching in zebrafish embryos. Kaplan−Meier hatching curves with 95% confidence intervals (shading) for embryos treated with Mmp13PI and controls. Hatch analysis indicated significant effects of treatment on hatching rate (*n* = 3, 20 embryos/replicate, *p* < 0.0001). Post-hoc pairwise comparisons identified significant effects among all treatments except between ERM and DMSO controls (*p* > 0.0001).

Pharmacological inhibition of Mmp13a has a significant effect on time to hatch in zebrafish embryos at 36 hpf (*p* < 0.0001) (Figure 4). At twice the IC_50_, the onset of hatching was delayed by over 12 h relative to control embryos, and only ~40% of embryos hatched by 72 hpf (compared to ~75%–80% in controls). At 10 times the IC_50_ value (100 nM), hatching was completely blocked. Embryos that failed to hatch at 100 nM were viable if manually removed from their chorions. None of these treatments are lethal and no morphological defects were observed. Pairwise comparisons using the Log-Rank test indicated that time to hatch for both concentrations of the inhibitor were significantly different from each other and controls (*p* < 0.0001 for all combinations), but there were no detectable effects on time to hatch between ERM and DMSO controls (*p* = 0.53).

### 3.3. Hypoxia-Induced Proteolysis in the Chorionic Fluid is Apparent at Both 24 and 36 hpf

**Figure 5:**
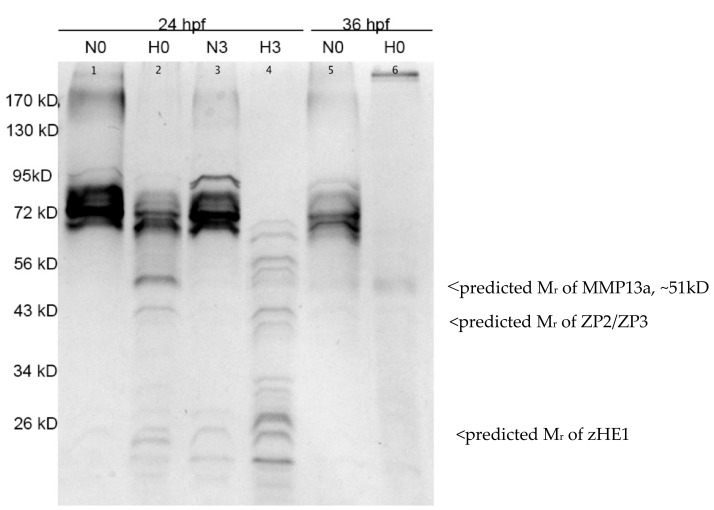
Environmental hypoxia induces proteolysis in the chorionic fluid of zebrafish embryos, as visualized by SDS-PAGE and silver stain. Samples in lanes 1-4 were collected from embryos treated at 24 hpf and lanes 5 and 6 from embryos treated at 36 hpf. N or H above the lanes denotes samples collected from normoxic controls or hypoxia treatments, respectively. The number indicates time elapsed in hours post treatment. Samples from lane 4 (H3) were collected from embryos 3 h after they were returned to normoxia following a 3 h hypoxia treatment that started at 24 hpf (therefore the embryos in N3 and H3 were 30 hpf). The expected relative mobility of active zebrafish Mmp13a, Zona pellucida 2 and 3, and zHE1 are indicated on the right.

Exposure to hypoxia (0.5% dO_2_, 0.4 mg/L) for 3 h triggers widespread proteolysis in the chorionic fluid (Figure 5). In embryos maintained in normoxia, we observe strong bands at molecular weights between ~70 and ~95 kD. In hypoxia-treated embryos, these higher molecular weight bands are less abundant, and we observe several bands appearing at lower molecular weights consistent with proteolysis. At 24 hpf, this effect is more pronounced 3 h after hypoxia treatment, as all of the bands above 72 kD are below the limit of detection and the lower molecular weight bands are stronger. At 36 hpf, this effect is even more dramatic with reduction of the high molecular weight bands even under normoxia. In the hypoxia-treated embryos at 36 hpf, the chorions were ragged leading us to suspect that the integrity of the chorion was already compromised and that proteins may have been leaking out. We could not collect chorionic fluid samples from 36 hpf embryos 3 h post-treatment as most of the embryos had hatched adding further support to this speculation.

### 3.4. Matrix Metalloproteinase 13 Activity in the Perichorionic Fluid of Embryos at 36 hpf

**Figure 6:**
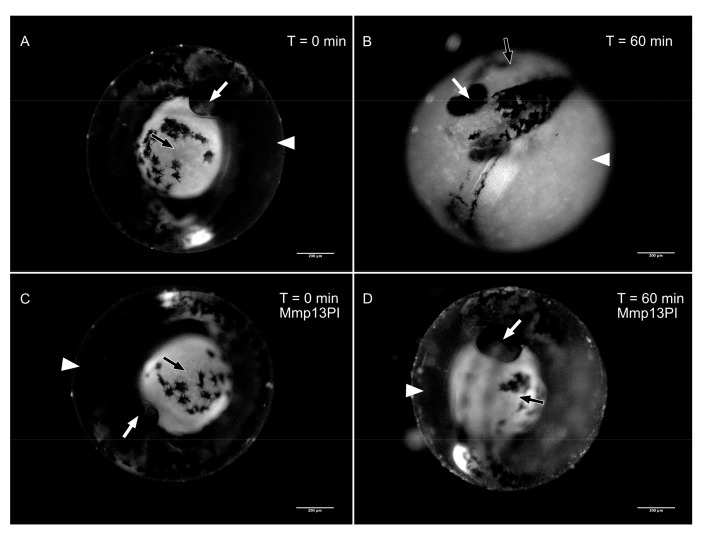
Mmp13a is active in the chorionic fluid of hatching-competent embryos. Epifluorescent micrographs of hatching-competent (36 hpf) embryos with Type I DQ Collagen injected into their chorionic fluid either without (**A**,**B**) or with (**C**,**D**) 20 nM Mmp13PI. Negligible fluorescent dequenching of the substrate is detectable immediately after injection (**A**,**C**), but in the absence of the inhibitor, the substrate is hydrolyzed over 1 h, generating a strong fluorescent signal in the peri-chorionic space (arrowheads). The eyes and yolk balls of the embryos are indicated with white and black arrows, respectively, for orientation. Scale bars are 200 µm. N = 10 for each treatment group.

Unfortunately, in our hands, the antibody against zebrafish Mmp13a did not label any bands on immunoblots of whole embryos or chorionic fluid; hence, we adopted an activity-based approach to infer the presence of Mmp13a in the chorionic fluid. Mmp13a is the only secreted MMP known to degrade fibrillar collagens encoded by the zebrafish genome [34]; hence, we used fluorogenic Type I DQ Collagen as a substrate for in vivo zymography [44,45]. Type I DQ Collagen injected into the peri-chorionic space of 36 hpf embryos undergoes rapid hydrolysis, resulting in fluorescent dequenching and increased fluorescent signal in the chorionic fluid (Figure 6A,B). Co-injecting this fluorogenic Mmp13 substrate with the Mmp13PI (20 nM) dramatically reduces dequenching of this Mmp13 substrate (Figure 6C,D).

## 4. Discussion

Adult animals experiencing environmental stress will typically alter their behavior to avoid stressors rather than compensating physiologically, as seen when Atlantic salmon (*Salmo salar*) aggregate at high density in cool thermal refugia when river temperatures get too high [51,52]. Behavior is inherently an emergent property of development that can only occur after organogenesis completes in the tissues relevant for that behavior. For instance, myogenesis and neurogenesis must occur before a swimming response, and the hatching gland must develop before hatching is possible. Prior to hatching, embryos cannot use locomotion to remove themselves from dangerous environments; metabolic compensation is their only option until reaching a developmental threshold referred to as “hatching competence”. This study demonstrates that pre-hatch embryos are not naïve to their environment, but their ability to respond appropriately depends on the developmental stage. The chorion is a poor barrier to abiotic factors such as temperature and dissolved oxygen; once hatching competence is achieved, pre-hatch zebrafish embryos growing under intolerably poor conditions can prematurely eject themselves from their sheltered chorionic space to face unknown conditions that might be more suitable for further development.

As reported previously, environmental hypoxia causes a slight developmental delay in zebrafish embryonic development [14,53]. In 24 hpf embryos, a 4 h hypoxic treatment is sufficient to significantly delay hatching (Figure 1, *p* < 0.01), whereas embryos exposed to hypoxia at 36 hpf hatch significantly earlier than normoxic controls (Figure 1, *p* < 0.001) despite the aforementioned developmental delay. Acute hypoxia is lethal at both developmental stages studied here if extended beyond 4 h (100% hatch, but 100% mortality at 6 h). The stress-induced hatch behavior has not emerged by 24 hpf; these embryos are trapped inside their chorion until they succumb to their fate. By 36 hpf, zebrafish embryos can A) detect environmental hypoxia, B) trigger the pathway responsible for activating the hatching enzyme, and C) physically break out of the chorion, sacrificing shelter for the potential of a better environment. It is still unclear which aspect(s) of this three-step hatching process is not competent at 24 hpf; we can only infer that hatching competence is achieved sometime between 24 and 36 hpf in zebrafish development. Owing to a dearth of knowledge regarding the molecular mechanism responsible for activating the hatching enzyme, we decided to pursue Yasumasu and colleagues’ supposition [25,27] that a metalloprotease in the hatching gland activates the hatching enzyme.

Mmp13a is already abundant in the hatching gland at 24 hpf and remains so until hatch, after which it disappears from this location, presumably due to its secretion from the hatching gland into the chorionic fluid. Inhibitor docking models comparing binding affinity indicate that the Mmp13PI will specifically target and inhibit zebrafish Mmp13a at concentration orders of magnitude lower than effective concentrations for inhibiting other proteases such as the HE itself [50], and targeted inhibition of Mmp13a activity dramatically inhibits hatching. Pharmacological MMP inhibitors have been studied extensively as potential drugs targeting cancer metastasis [54] and other disease pathologies [55,56], but limited progress using broad-spectrum inhibitors has both emphasized the importance of nuance in MMP biology and driven the development of highly-specific inhibitors to target individual MMPs. Despite the fact that this molecule was developed to target human MMP-13 rather than zebrafish Mmp13a, it has been successfully used experimentally in zebrafish [39,57] and qualitatively produces phenotypic effects analogous to effects observed in mammals. The absence of apparent morphological defects when embryos are reared in Mmp13PI is surprising, as Mmp13a is not restricted to the hatching gland (Figure 2) [38,39,57] and morpholino knockdowns of Mmp13a are embryonic lethal with craniofacial deformities and extreme spinal curvature [38]. The reported effects of Mmp13PI in vivo are more subtle; for example, Mmp13PI protects sensory neurons in the ectodermal epithelia of zebrafish from multiple models of neurotoxicity [39,57]. This suggests that the disconnect between the phenotype of *mmp13a* morphants and Mmp13PI-inhibited embryos is due to limited penetrance of the drug into tissues; phenotypic effects are observed in the epithelium (at µM concentrations) and in the chorionic fluid (at nM concentrations) (Figure 3, Figure 5 and Figure 6), but not deeper inside the tissues of the embryo. Here, we provide in silico evidence that the effective concentration of the Mmp13PI for zebrafish Mmp13a, may be slightly higher than the empirically determined value for human MMP-13 (20 vs. 8 nM) but is still far below concentrations where we would expect to see off-target effects on other MMPs (µM concentrations), let alone the astacin-like HE. Taken together, these data suggest that Mmp13a activity is a necessary component of the HE activation system.

Hypoxia induces widespread proteolysis in the chorionic fluid of zebrafish embryos at both time points examined in this study. At 24 hpf and 36 hpf (both prior to normal hatching times), the chorionic fluid contains large proteins, but we observe the loss of these high molecular weight proteins and the appearance of smaller fragments after hypoxia treatment. This assay of chorionic protein composition provides a qualitative view of overall proteolysis but does not specifically implicate Mmp13a. Mmp13a activity was assayed specifically using a fluorogenic substrate for collagenases (DQ Type I Collagen) combined with specific pharmacological inhibition of Mmp13a, which blocks the hydrolysis of this Mmp13 substrate. This is not to suggest that Mmp13a is normally breaking down collagen in the chorionic fluid or chorion as there is no evidence of collagen being present in these matrices. MMPs are not restricted to a single substrate [28]; the collagenolytic nature of the chorionic fluid acts as a reporter for the activated, uninhibited Mmp13a protein acting on some unknown substrate (perhaps HE). Proteolysis of chorionic proteins by itself does not completely explain hatching competence, as it also occurs at 24 hpf, before embryos can hatch in response to hypoxia. However, chorionic fluid is highly collagenolytic at the onset of hatching competence, and inhibition of Mmp13a activity prevents hydrolysis of exogenously provided collagen and hatching under standard conditions, as well as stress-induced hatching.

Taken together, these experiments suggest a model for hatching in which large proteins present in the chorionic fluid prior to hatch are proteolyzed into smaller fragments as the proteolytic hatching mechanism becomes active, regardless of whether other necessary components of the hatching system are active. Mmp13a is necessary for collagenolytic activity in the chorionic fluid and for hatching itself, but clearly, this proteolysis is not sufficient to explain why zebrafish embryos are hatching competent at 36 hpf, but not at 24 hpf. We posit that the ability to hatch prematurely in response to poor environmental conditions confers an advantage to embryos, as it enables them to escape these stressors rather than attempt to compensate for them. This idea is supported by the observation that chorionic membranes of Atlantic salmon eggs from high flow rivers are more porous than those from low flow rivers and that porosity (and, by proxy, oxygen diffusion into the embryo) was the best predictor of hypoxia tolerance of pre-hatch embryos [58]. Population-specific differences in egg membrane structure linked to hypoxia tolerance indicate that the interaction between environmental oxygen availability and pre-hatch embryogenesis is adaptive. If this interaction is indeed adaptive, we would predict that the different populations of Atlantic salmon described in Bloomer et al. [58] would have different dO_2_ “trigger levels” to induce hatching depending on porosity. It follows then that if embryos have not developed locomotory function, they will not be able to take advantage of this mechanism. The ontogeny of swimming behavior in hatched zebrafish has been described in detail starting at 48 hpf [59]. These early larvae are poor swimmers due to the relatively large size of their yolk ball and it seems reasonable to assume that this would be exacerbated at younger stages. We are unaware of any studies that have measured swimming behavior in zebrafish embryos earlier than 48 hpf, but if 36 hpf embryos are significantly better swimmers than 24 hpf embryos, then perhaps this explains why the former can hatch but the latter cannot. Most teleosts can swim immediately upon hatch [60]; perhaps this is because the emergence of swimming behavior is a necessary component of hatching competence.

## 5. Conclusions

The identification of Mmp13a as an integral regulator of hatching in zebrafish embryos emphasizes the fact that spatiotemporal localization of zymogens (let alone transcripts) is not sufficient for inferring biological activity; it is equally important to know when and where the activators are present. Mmp13a is the first protein identified in the upstream activation pathway of zHE1, but it is not yet clear if Mmp13a is activating zHE1 directly or if it is further upstream. Delving into the specific biochemistry of zHE1 activation will require an antibody against zHE1 or some other technique to measure metalloprotease activation such as the Epitope-Mediated MMP Activation assay [33,61]. Identification of Mmp13a as the putative activator of zHE1 has, in a sense, moved the goalposts, as now we must ask how Mmp13a is being activated and regulated in the hatching gland. Elucidation of the hatching enzyme activation mechanism is critical for understanding how animals escape from the confines of their chorion and how this process is modulated by the environment.

It will be interesting to compare this mechanism among species of different taxa across the animal kingdom. Even among teleosts, there is variation, as many species have evolved a hatching mechanism involving HE1 and the paralogous HE2 [62]. In human development, pre-implantation hatching has been described as the “black box” of early pregnancy loss [63]. Mouse models developed to study implantation and early mammalian development emphasize the importance of the synchronization of embryonic development and uterine receptivity; embryos must achieve “implantation competence”, a process dependent on selective proteolysis by unknown proteases [64]. Despite a detailed understanding of the cell biology of the early mammalian embryo [65,66] and implantation [64,67], blastocyst hatching has not been studied extensively. Implantation rates are improved in a delayed implantation model that extends blastocyst quiescence prior to implantation [68], perhaps by providing more time for the embryo to express and activate the proteases necessary to digest the ZP and/or ECM of the uterine lining. Current standard practice for human in vitro fertilization is to transfer expanded blastocysts after in vitro culture [65]; however, even expanded blastocysts often fail to hatch after transfer [68]. Assisted-hatch techniques such as zona-scratching to improve in vivo hatching have shown promising but inconsistent results [69]. By extending blastocyst culture time and transferring only spontaneously hatched blastocysts, Chimote and colleagues improved implantation success and live birth rate, thereby demonstrating that successful hatching is a better prognostic marker for in vitro fertilized embryo selection than blastocyst expansion [68]. Although hatching itself is ubiquitous across taxa, it is unclear if the molecular mechanisms of hatching are conserved. The zebrafish is unlikely to be a useful model to study uterine implantation. However, the mechanism(s) regulating hatching are likely highly conserved, and the zebrafish embryo provides an excellent model in which to study these processes.
